# Long noncoding RNA GAPLINC promotes invasion in colorectal cancer by targeting SNAI2 through binding with PSF and NONO

**DOI:** 10.18632/oncotarget.9741

**Published:** 2016-05-31

**Authors:** Peng Yang, Tao Chen, Zipeng Xu, Hua Zhu, Jie Wang, Zhenyu He

**Affiliations:** ^1^ The Second Clinical Medical College of Nanjing Medical University, Nanjing, China; ^2^ Department of General Surgery, The Second Affiliated Hospital of Nanjing Medical University, Nanjing, China

**Keywords:** colorectal cancer, GAPLINC, long noncoding RNA, NONO, PSF

## Abstract

This study aimed to investigate the role of long noncoding RNAs (lncRNAs) in the metastasis of colorectal cancer (CRC). Metastasis is an important prognostic factor of CRC, and lncRNAs have been implicated in tumor proliferation and metastasis. The human CRC cell lines HCT116, HT29, SW480, DLD-1, and SW620 were used in the study. Genome-wide lncRNA expression patterns in metastatic lymph nodes compared with paired normal lymph nodes of CRC were assessed by microarray analysis. Gastric adenocarcinoma predictive long intergenic noncoding (GAPLINC) RNA was detected via functional prediction. The increased expression of GAPLINC was found to be positively correlated with larger tumor size, advanced tumor stage (T stage), advanced node stage (N stage), increased death, and shorter survival of patients with CRC by *in situ* hybridization analysis. Besides, the decreased expression of GAPLINC could significantly repress CRC cell invasion *in vitro* and also inhibit proliferation *in vitro* and *in vivo*. RNA pull-down with mass spectrum experiments revealed that PTB-associated splicing factor (PSF) and non-POU-domain-containing octamer-binding (NONO) protein bound to GAPLINC and reversed the effect of GAPLINC on cell invasion. Gene array and bioinformatics analyses identified that snail family zinc finger 2 (SNAI2) was involved in the biological processes of GAPLINC/PSF/NONO. This study indicated the importance of GAPLINC in promoting CRC invasion via binding to PSF/NONO and partly by stimulating the expression of SNAI2. Hence, GAPLINC may serve as a promising target for CRC diagnosis and therapy. The findings may help in developing a novel therapeutic strategy for patients with CRC.

## INTRODUCTION

Colorectal cancer (CRC) is the third most commonly diagnosed cancer and the fourth most common cause of cancer deaths worldwide, with over 1.2 million new cases each year [1, 2]. Clinically, a considerable number of patients with CRC develop metastases later, such as distant metastasis and lymph node metastasis, resulting in a relatively high overall mortality rate of 40%–45% [3]. Genes participating in the CRC metastasis process have been widely investigated. A previous study used clinical CRC samples and investigated metastatic activities in a mouse rectal transplantation model *in vivo*. The study reported that chemokine (C-X-C motif) receptor 3 (CXCR3) and chemokine (C-X-C motif) receptor 4 (CXCR4) were involved in the metastasis of CRC to lymph nodes, lungs, and liver. Thus, targeting CXCR3 and CXCR4 could be a promising therapy against CRC metastasis [4].

Long noncoding RNAs (lncRNA), nonprotein coding transcripts more than 200 nucleotides in length, have been also implicated in tumor proliferation and metastasis [5–7]. For instance, long noncoding RNA HOX antisense intergenic RNA (HOTAIR), closely associated with metastasis, epithelial–mesenchymal transition (EMT), and poor prognosis, has emerged as a key metastatic molecule in many cancers [8–10]. The metastasis-associated lung adenocarcinoma transcript 1 (MALAT1), the first lncRNA identified as an independent prognostic marker for nonsmall-cell lung cancer [11], was found to play an important role in the metastasis of various types of cancers [12–14]. However, the roles of lncRNAs in the complex progression of CRC metastasis need to be further studied.

Lymph node is the most common metastatic site with a nearly 50% disease recurrence rate [15]. To determine whether certain lncRNAs are involved in lymph node metastasis of CRC, lncRNAs in metastatic lymph nodes (MLNs) and normal lymph nodes (NLNs) of CRC were screened based on lncRNA microarray. In this study, a long intergenic noncoding RNA (lincRNA) named gastric adenocarcinoma predictive long intergenic noncoding (GAPLINC) RNA was identified, which was significantly associated with the poor prognosis of CRC. GAPLINC was found to combine with PTB-associated splicing factor (PSF) and non-POU-domain-containing octamer-binding (NONO) protein. These were necessary for the effect of GAPLINC in promoting colorectal cancer cell invasion partly via increasing the expression of snail family zinc finger 2 (SNAI2).

## RESULTS

### GAPLINC was involved in CRC metastasis

**Figure 1:**
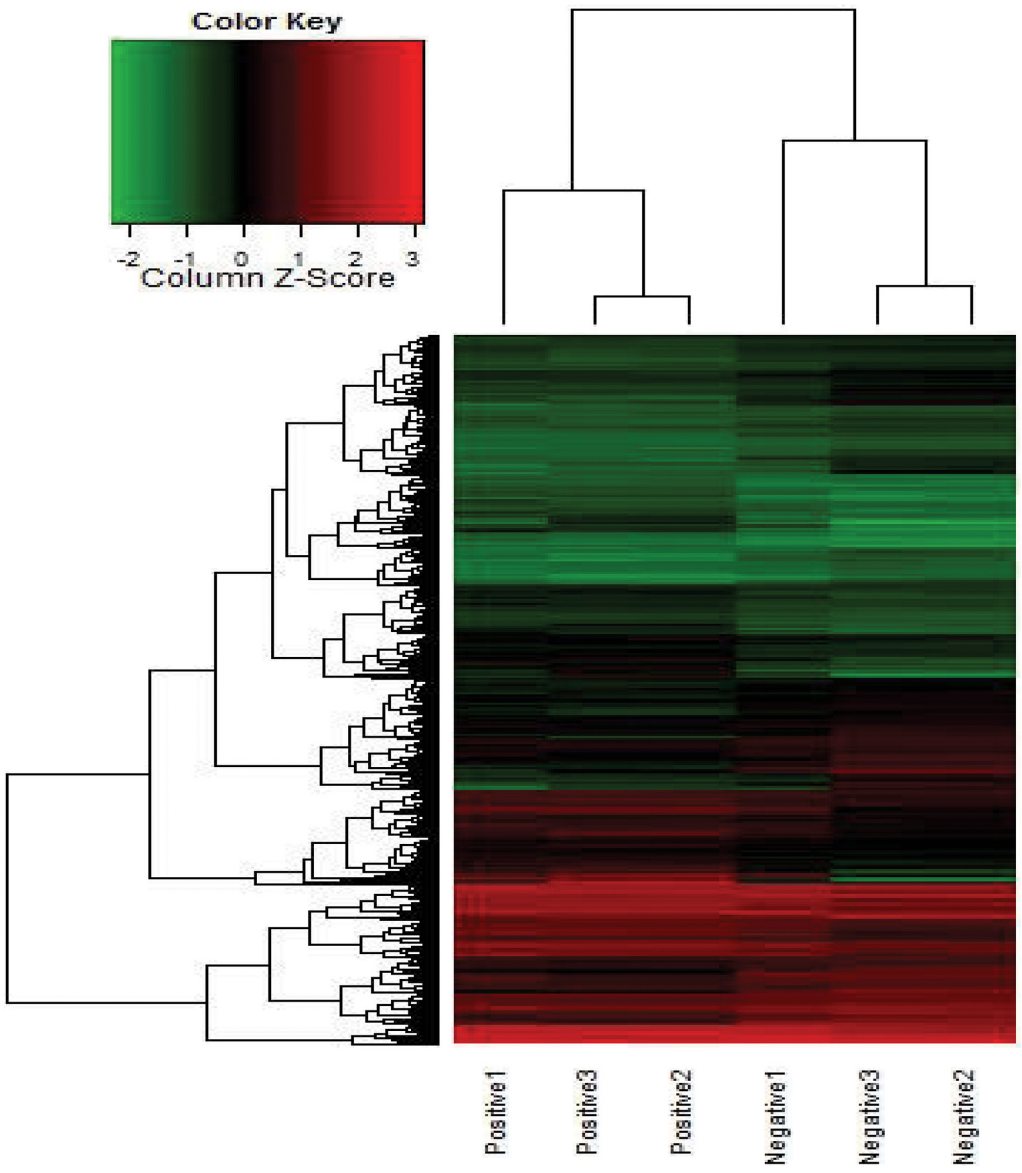
Heat map for the co-expression mRNAs of GAPLINC expressed in MLNs compared with NLNs Each row represents one mRNA, and each column represents one sample. Red indicates an increase and green indicates a decrease in relative mRNA expression.

To search for potential lncRNAs involved in the metastatic progression of CRC, the lncRNA expression in MLNs and NLNs from three CRC patients were profiled using the Human LncRNA Array. A total of 1133 lncRNA transcripts (fold change ≥2.0, *P* ≤ 0.05) were dysregulated in MLNs and compared with NLNs. Then, a heat map was generated to describe the differentially expressed lncRNAs and mRNAs in Supplementary Figure S1. Among these dysregulated lncRNA transcripts, a long intergenic noncoding RNA (lincRNA) uc002kmd.1, located at 18p11.31, was detected, which had 9.12-fold expression in MLNs than in NLNs. The Pearson correlation of the expression value of GAPLINC with the expression value of each mRNA was calculated, and a heat map was built to describe the relationships between GAPLINC and its co-expression mRNAs (Figure 1). The results of the pathway analyses using Kyoto Encyclopedia of Genes and Genomes (KEGG) annotations based on these co-expression mRNAs showed that lincRNA-uc002kmd.1 was associated with the pathogenesis of CRC, involving cell adhesion molecules, Wnt signaling pathway, NF-kappa B signaling pathway, and adherens junction (Supplementary Table S2). LncRNA-uc002kmd.1, also named GAPLINC, was reported to be associated with CD44-dependent cell invasiveness and poor prognosis of gastric cancer [16].

### Expression of GAPLINC was associated with the poor prognosis of CRC

**Figure 2:**
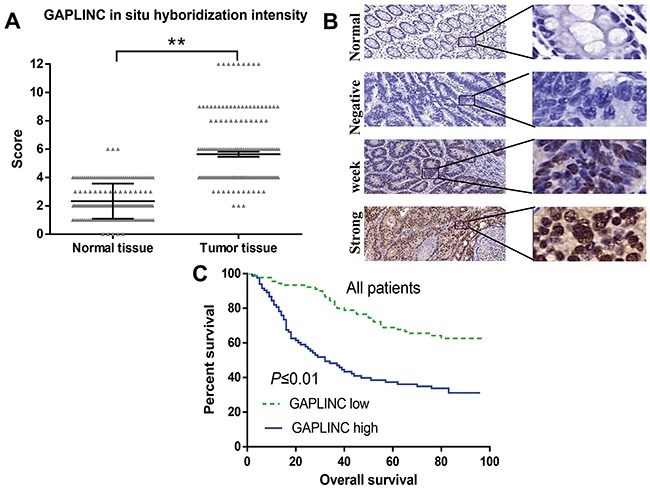
GAPLINC expression correlated with worse prognosis for patients with CRC **A.** GAPLINC significantly increased in 180 human CRC tissues compared with the adjacent normal tissues (*P* < 0.01). The *Y*-axis indicates the staining score (intensity*area) of GAPLINC. **B.** Representative ISH characteristics of GAPLINC expression are shown (magnification, left panel, ×100; right panel, ×200). **C.** Kaplan–Meier survival curves revealed an association of higher GAPLINC levels with a short OS of patients with CRC. Data represent mean ± standard deviation from three independent experiments. *P < 0.05, **P < 0.01.

To explore whether GAPLINC expression was associated with the poor prognosis of CRC cancer, ISH was used to assess the expression level of GAPLINC in 180 pairs of CRC tissues and matched normal tissues. The clinical pathological characteristics of the 180 patients with CRC are summarized in Table 1. The expression level of GAPLINC was markedly higher in cancer tissues compared with normal tissues (Figure 2A), as representatively shown in Figure 2B. The correlation analysis of GAPLINC expression revealed a significant association between GAPLINC expression and CRC tumor size (*P* < 0.01), tumor stage (T stage) (*P* < 0.01), node stage (N stage) (*P* < 0.01), and increased death (*P* < 0.01) (Table 2). However, the association between GAPLINC expression and patient age, gender, and grade was not found. Kaplan–Meier survival analysis and log-rank tests revealed that patients with high GAPLINC expression had a significantly shorter overall survival (OS) than those with low GAPLINC expression (Figure 2C, *P* < 0.01). Univariate and multivariable Cox regression analyses revealed that grade and high GAPLINC expression were risk factors for the OS of patients with CRC (Table 2). These results indicated that GAPLINC expression could be an independent factor for increasing the OS.

**Table 1:** Association of GAPLINC expression with clinical pathological parameters of patients with CRC

Parameters	*N*	GAPLINC expression		*P* value
		Low	High	
**Age (year)**				0.881
**≤60**	39	21	18	
**>60**	141	74	67	
**Sex**				0.644
**Female**	88	48	40	
**Male**	92	47	45	
**Tumor size (cm)**				<0.01**
**≤5**	101	70	31	
**>5**	77	23	54	
**T stage**				<0.01**
**1**	13	9	4	
**2**	96	57	39	
**3**	66	27	39	
**4**	3	1	2	
**N stage**				<0.01**
**N0**	111	67	44	
**N1**	51	24	27	
**N2**	18	4	14	
**Grade**				0.116
**I**	32	18	14	
**II**	139	76	63	
**III**	9	1	8	
**Death**				<0.01**
**No**	85	57	28	
**Yes**	89	33	56	

* Statistically significant (*P* < 0.05).

**Table 2:** Univariate and multivariate Cox regression analyses of GAPLINC expression and clinical variables for overall survival of patients with CRC

Variables	Categories	Univariate analysis			Multivariate analysis		
		HR	95% CI	*P* value	HR	95% CI	*P* value
Age (year)	>60/≤60	1.514	0.869–2.637	0.143			
Sex	Maleemale	0.974	0.642–1.475	0.905			
Size (cm)	≥5/<5	1.519	1.002–2.303	0.045*	1.156	0.728–1.834	0.539
T stage	1/2/3/4	2.153	1.534–3.021	<0.01**	1.222	0.668–2.233	0.516
N stage	N_0_/N_1_/N_2_	2.144	1.615–2.846	<0.01**	1.528	0.900–2.595	0.116
Grade	I/II/III	2.789	1.575–4.940	<0.01**	1.972	1.197–3.250	<0.01**
GAPLINC	High / low	2.730	1.771–4.207	<0.01**	2.214	1.375–3.565	<0.01**

Abbreviations: HR, hazard ratio; 95% CI, 95% confidence interval.

* Statistically significant (*P* < 0.05).

### GAPLINC promoted cell proliferation and invasion *in vitro*

**Figure 3:**
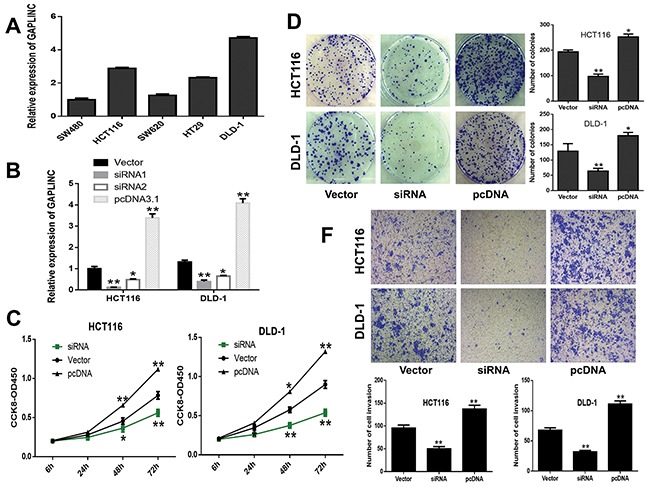
GAPLINC promoted CRC cell proliferation and invasion *in vitro* **A.** Relative expression level of GAPLINC in CRC cell lines. **B.** GAPLINC mRNA levels of empty vector, GAPLINC-siRNA1, GAPLINC-siRNA2, and GAPLINC-pcDNA3.1 by qRT-PCR in HCT116 and DLD-1 cells. **C, D.** CCK-8 viability and colony-forming growth assays showed that HCT116 and DLD-1 cells transfected with siRNA1 inhibited the cell proliferation of CRC cells and increased the proliferation with GAPLINC-pcDNA3.1. **E.** Transwell assay showed that the knockdown of GAPLINC significantly suppressed cell invasion ability and upregulated GAPLINC-enhanced invasion. Data represent mean ± standard deviation from three independent experiments. *P < 0.05, **P < 0.01.

Attempts were made to investigate the effects of GAPLINC on CRC cells. First, the expression level of GAPLINC was detected in CRC cell lines, SW480, SW620, HCT116, HT29, and DLD-1. GAPLINC expression was found to be significantly higher in HCT116 and DLD-1 compared with other CRC cell lines (Figure 3A). Then, two specific siRNAs and pCDNA3.1 vector were transiently transfected into HCT116 and DLD-1 cells (Figure 3B), and siRNA1 demonstrated the highest silencing capacity, which was chosen for subsequent experiments. After transfection for 48 h, the CCK-8 and colony formation assays indicated that the decrease in GAPLINC could significantly inhibit cell proliferation and clonogenic survival in both HCT116 and DLD-1 cell lines compared with the control cells, while the overexpression cells showed the opposite effect (Figure 3C and 3D). Next, the effects of GAPLINC on invasion were investigated. The results of Transwell assay showed that the silencing of GAPLINC could significantly impair CRC cell invasion ability compared with control cells and that the GAPLINC-pcDNA3.1 could strengthen the ability (Figure 3E).

### GAPLINC promoted CRC cell proliferation *in vivo*

**Figure 4:**
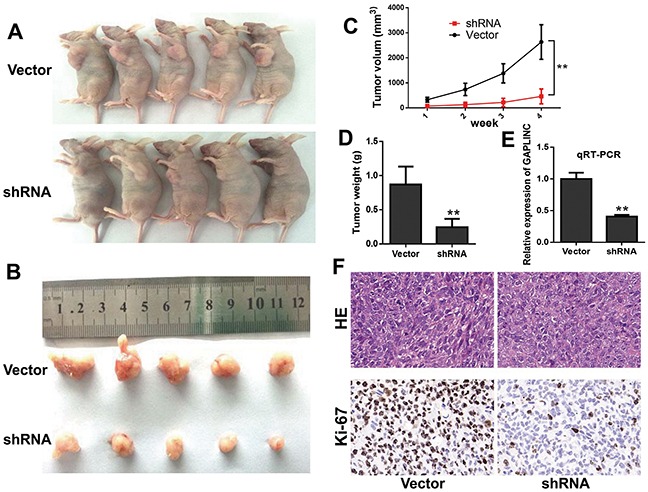
GAPLINC accelerated the growth of CRC cells *in vivo* **A, B.** Photographs of the xenograft-transplanted nude mouse tumor models and the harvested tumors were captured 4 weeks after the injection of GAPLINC-shRNA or empty vector of HCT116 cells (*n* = 5). **C.** Tumor volume was calculated as the length × width^2^ × 0.5 every week after injection. **D.** Weights of xenografts were measured when the mice were sacrificed. **E.** qPCR analysis of GAPLINC expression in xenograft tumor tissues. **F.** Hematoxylin and eosin and immunohistochemical (IHC) staining of the xenograft tumors. IHC staining showed that Ki67 expression was weakened in the GAPLINC-shRNA group compared with the empty vector group. Data represent mean ± standard deviation from three independent experiments. *P < 0.05, **P < 0.01.

To further examine whether GAPLINC mediated tumorigenesis *in vivo*, HCT116 cells transfected with GAPLINC-shRNA or empty vector were injected into the male nude mice. As a result, both the growth and the volume of tumor xenografts significantly decreased in the GAPLINC-shRNA groups compared with the control groups (Figure 4A–4D). The qRT-PCR analysis demonstrated that the average expression levels of GAPLINC in the interference groups were lower than those in the empty groups (Figure 4E). The expression of proliferation index Ki-67 significantly decreased in the GAPLINC-shRNA transfected tumors, as detected by immunochemical analysis (Figure 4F). These results revealed that the knockdown of GAPLINC could repress CRC cell proliferation *in vivo*.

### GAPLINC regulated cell invasion through PSF and NONO

**Figure 5:**
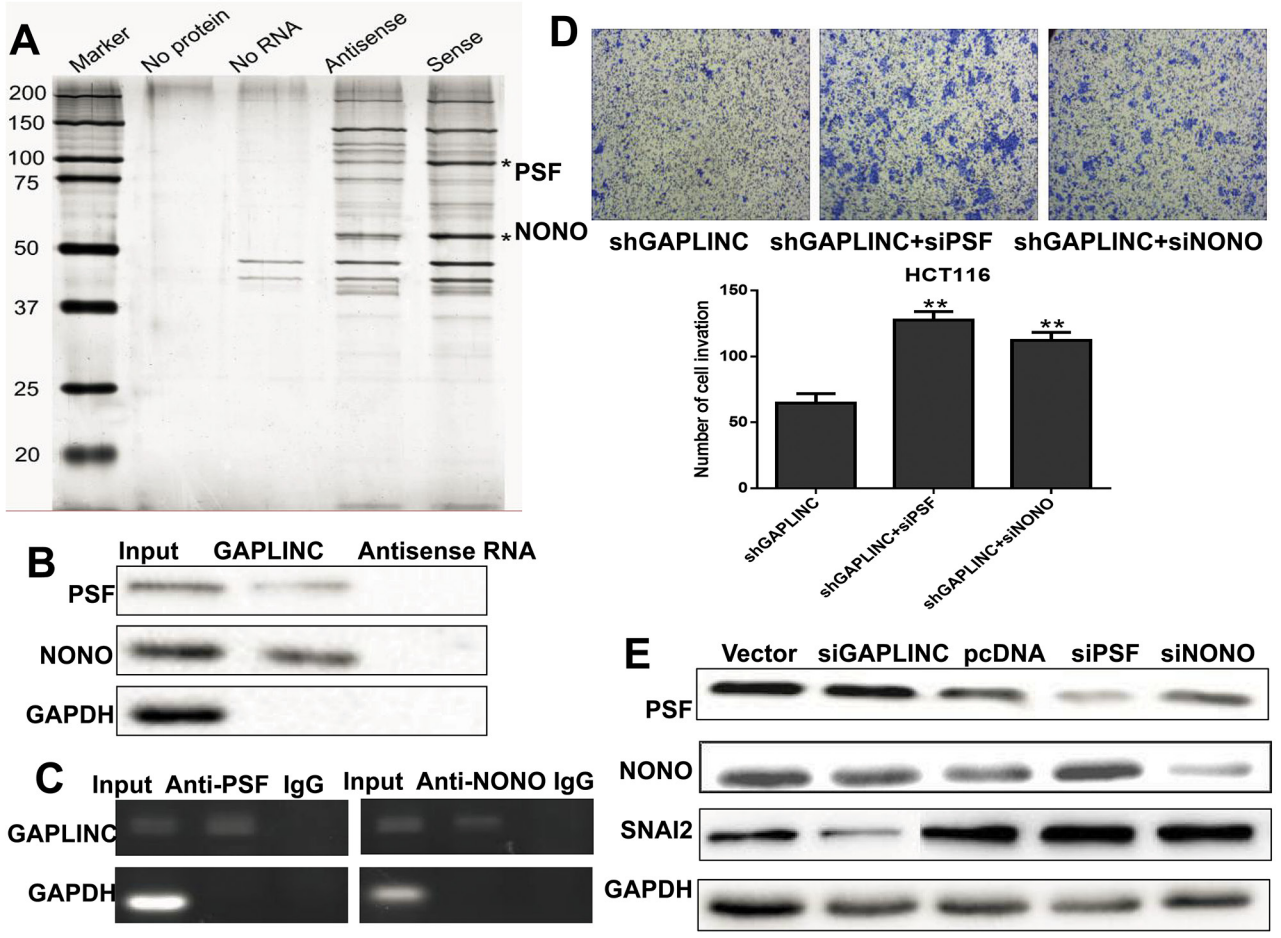
GAPLINC bound to PSF and NONO **A.** RNA pull-down was performed using a GAPLINC template and RNA-bound protein separated by SDS-PAGE in HCT116. The protein bands were excised and detected by mass spectrometry analysis. **B.** PSF and NONO were detected by the Western blotting assay in the samples pulled down by GAPLINC. **C.** RIP analyses were performed using antibodies against PSF and NONO, with IgG as a negative control in HCT116. The enrichment of the GAPLINC was detected using RT-PCR and normalized to the input. **D.** The Transwell assay was performed to assess the invasion ability of HCT116-GAPLINC-shRNA cells transiently transfected with empty vector, PSF-siRNA, and NONO-siRNA. **E.** Western blotting analysis showed that the protein levels of PSF, NONO, SNAI2, and GAPDH were detected in HCT116 empty vector, GAPLINC-siRNA, GAPLINC-pcDNA3.1, PSF-siRNA, and NONO-siRNA cells. Data represent mean ± standard deviation from three independent experiments. *P < 0.05, **P < 0.01.

Several recent studies have shown that many lncRNAs could participate in molecular regulation pathways via their binding with proteins. To investigate whether GAPLINC functioned in a similar manner, RNA pull-down assays were performed to identify proteins associated with GAPLINC in HCT116 cells (Figure 5A). PSF and NONO were the main proteins identified by mass spectrometry (Supplementary Table S3), and both of them were detected by Western blotting in three independent RNA pull-down assays (Figure 5B). RIP was also performed using anti-PSF and anti-NONO (IgG for control) in cell extracts from HCT116 cells. GAPLINC enrichment with PSF and NONO antibodies was observed, but not GAPDH mRNA enrichment (Figure 5C). These results suggested that GAPLINC probably physically bound with both PSF and NONO.To characterize the effects of GAPLINC on PSF and NONO, it was found that the effect of GAPLINC-shRNA on cell invasion could be neutralized by the knockdown of PSF or NONO using the Transwell assay (Figure 5D). These findings suggested that PSF and NONO were necessary for the effects of GAPLINC in CRC cells. However, qRT-PCR and Western blotting were performed to reveal that the expression levels of PSF and NONO remained unaltered in GAPLINC-overexpressing and GAPLINC-knockdown HCT116 cells, as shown in Figure 5E (mRNA data not shown).

### SNAI2 was a target of GAPLINC

**Figure 6:**
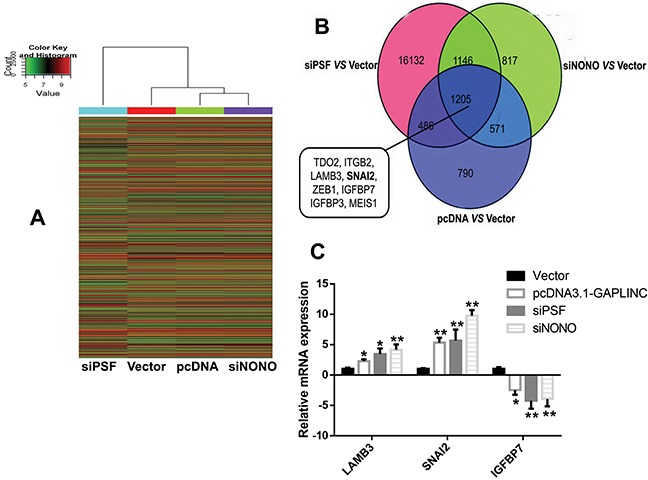
Microarray analysis investigating the GAPLINC-associated genes **A.** Heat map image of different gene expression in the HCT116 cells transfected with empty vector, GAPLINC-pcDNA3.1, PSF-siRNA, and NONO-siRNA. Red indicates an increase and green indicates a decrease. **B.** Venn diagram of genes with altered gene expression as identified using microarray analysis. Representative genes found in the overlap between these three groups are displayed. **C.** RT-qPCR was used to detect the mRNA levels of LAMB3, SNAI2, and IGFBP7 in the HCT116 cells treated with empty vector, GAPLINC-pcDNA3.1, PSF-siRNA, and NONO-siRNA. Data represent the mean ± standard deviation from three independent experiments. *P < 0.05, **P < 0.01.

To probe the GAPLINC-associated pathway, the microarray analysis revealed 3052 GAPLINC-inducible genes through the increase in GAPLINC in HCT116 cells (Figure 6A). The induction of 1691 of these GAPLINC-inducible genes was overlapped by PSF knockdown. Moreover, 1776 genes were identified that were overlapped via NONO knockdown (Figure 6B). Many tumor metastasis genes, such as *tryptophan 2,3-dioxygenase* (*TDO2*), *integrin subunit beta 2* (*ITGB2*), *laminin, beta 3* (*LAMB3*), *SNAI2*, *zinc finger E-box binding homeobox 1* (*ZEB1*), *insulin-like growth factor–binding protein 7* (*IGFBP7*), *insulin-like growth factor–binding protein 3* (*IGFBP3*), and *Meis homeobox 1* (*MEIS1*), were identified in the overlap of these three groups, and the mRNA expression levels of *LAMB3*, *SNAI2*, and *IGFBP7* were verified by RT-qPCR assays (Figure 6C). The knockdown of GAPLINC also decreased the expression of SNAI2 by Western blot analysis (Figure 5E). These results suggested that PSF and NONO were involved in the effects of GAPLINC partly via upregulating SNAI2.

## DISCUSSION

Nowadays, the percentage of patients with CRC metastasis may reach up to 30%, with a 5-year survival rate lower than 10% [17, 18]. Therefore, it is significant to investigate the genetic changes and underlying mechanisms of CRC metastasis. The present study focused on a lincRNA named GAPLINC. The lncRNA array and bioinformatics analysis revealed that GAPLINC displayed a higher expression level in MLNs compared with NLNs of CRC. Attempts were made to evaluate the relationship between GAPLINC expression and prognosis of 180 patients with CRC using TMA and ISH methods. GAPLINC was found to be expressed at a higher level in CRC tissues than in adjacent normal tissues and was significantly associated with tumor size, T stage, N stage, increased death, and shorter survival time. These results showed that GAPLINC might exhibit an important role in CRC carcinogenesis.

The potential role of GAPLINC in the biological processes of CRC cells was also evaluated. The functions of CRC cell proliferation and invasion were disturbed by siRNA-mediated knockdown. GAPLINC-shRNA were found to repress CRC cell tumorigenicity *in vivo*. These observations were in agreement with the results of a study on gastric cancer [16].

Recent studies have demonstrated that many lncRNAs can participate in cancer development by binding with proteins. For instance, lncRNA-hPVT1, by binding to the RNA-binding protein NOP2, was found to increase NOP2 levels by enhancing the stability of NOP2 proteins and hence promote carcinogenesis, cell proliferation, and stem cell-like properties in hepatocellular carcinoma cells depending on the presence of NOP2 [19]. In this report, PSF and NONO were identified as the binding protein of GAPLINC by RNA pull-down assay and RNA-binding protein immunoprecipitation.

PSF (also known as SFPQ) is a multifunctional protein involved in transcription regulation, pre-mRNA splicing, and DNA repair [20, 21]. A study revealed that PSF was a putative tumor-suppressor protein, containing an RNA-binding domain and a DNA-binding domain [22]. Ji et al found that MALAT1 could competitively bind to PSF to promote CRC cell proliferation and migration by releasing PTBP2 from the PSF/PTBP2 complex. Moreover, PSF plays a key role in the regulatory effect of MALAT1 on cell proliferation and migration [23]. NONO (also named p54nrb) may also be implicated in cancer because of its roles in many biological processes, including RNA splicing, DNA repairing, and gene transcription [24, 25]. A recent study demonstrated that lncUSMycN RNA bound to NONO, leading to an increase in N-Myc RNA and proliferation of neuroblastoma cells [26]. The present study found that both PSF and NONO could neutralize the effect of GAPLINC on cell invasion. However, the mRNA and protein levels of PSF and NONO did not change when interfering with the expression of GAPLINC. These results implied that PSF and NONO might act as special regulators to affect the biological functions of GAPLINC by mediating other factors.

Increasing data reported that PSF could form a heterodimer with NONO, and they often both increased or decreased gene transcription [27, 28]. The GAPLINC-associated genes (e.g., SNAI2) that were also affected by PSF and NONO were surveyed using microarray analysis. SNAI2, a member of the snail family of transcription factors, can promote cell invasion, motility, metastasis, and poor prognosis via inhibiting the E-cadherin transcription and inducing EMT in several human cancers [29–31]. This study demonstrated that PSF and NONO were involved in the biological processes of GAPLINC; however, how these two proteins bound with GAPLINC and how they activated the GAPLINC-associated genes to promote invasion still needed to be further explored.

In summary, the results showed that GAPLINC was a potential prognostic factor for patients with CRC and regulated the biological functions of CRC cells. GAPLINC was also found to combine with PSF and NONO to promote the influence of GAPLINC on cell invasion partly via increasing the expression of SNAI2. The present findings may help in better understanding the lncRNA/PSF/NONO binding and developing a novel therapeutic strategy for patients with CRC.

## MATERIALS AND METHODS

### Cell lines and cell culture

The human CRC cell lines HCT116, HT29, SW480, DLD-1, and SW620 were cultured in Dulbecco's modified Eagle medium (Gibco, NY, USA) supplemented with 10% fetal bovine serum (FBS) (Gibco) and 1% penicillin–streptomycin in a humidified atmosphere containing 5% CO_2_ at 37°C.

### Tissue microarray and *in situ* hybridization

Two tissue microarrays (TMAs) containing 180 pairs of CRC tissues and normal tissues were purchased from Biochip Company (Shanghai, China) (Catalog no. HCol-Ade180Sur-04 and HCol-Ade180Sur-08). *In situ* hybridization (ISH) was carried out on 4-μm-thick TMA sections. First, the sections were fixed and permeabilized to allow access to GAPLINC probes, and then boiled in a pretreatment buffer. Hybridization was performed overnight after the GAPLINC probes were denatured. Finally, the lncRNA was visualized with the enzymatic reaction of 3,3′-diaminobenzidine, and the sections were counterstained with hematoxylin.

Two pathologists who were blinded to the clinical data evaluated the stained sections according to the German immunoreactive score (IRS). Staining intensity was assigned a score as “0 = negative,” “1 = weak,” “2 = moderate,” and “3 = strong”; staining extent was assigned a score as “0 = <5%,” “1 = 5%–25%,” “2 = 25%–50%,” “3 = 50%–75%,” and “4 = >75%.” By multiplying the intensity score and the extent score (from 0 to 12), the final IRS of GAPLINC expression was generated. For each section, the median score ≥6 was defined as high GAPLINC expression and the rest as low GAPLINC expression.

### SiRNA and plasmid transfection

The siGAPLINC, siPSF, siNONO, and negative control were synthesized by Invitrogen (Shanghai, China) with the siRNA sequences listed in Supplementary Table S1. The GAPLINC full-length sequence was synthesized and subcloned into a pCDNA3.1 vector (Invitrogen, Shanghai, China). The cells were transfected with the aforementioned siRNA and pCDNA3.1 vectors for 48 h using Lipofectamine 3000 (Invitrogen, Shanghai, China) following the manufacturer's protocol. The efficiency of knockdown and overexpression were determined by real-time quantitative reverse transcription-polymerase chain reaction (qRT-PCR).

### Quantitative real-time polymerase chain reaction

Total RNA was extracted from the cells and tissues using Trizol reagent (Invitrogen, Shanghai, China), and then reverse transcribed to cDNA with PrimeScript RT polymerase (Takara, Tokyo, Japan). All the primer sequences are shown in Supplementary Table S2. For RT-PCR, the products were confirmed with agarose electrophoresis. Real-time PCR was performed using SYBR Green (Takara, Japan) according to the manufacturer's instructions. Glyceraldehyde-3-phosphate dehydrogenase (GAPDH) was used as an internal control for normalization.

### Cell proliferation

The Cell Counting Kit 8 (CCK-8, Dojindo, Kumamoto, Japan) was used to assess the relative cell viability at 24, 48, and 72 h after transfection according to the manufacturer's instruction. As for the colony formation assay, a total of 500 cells were seeded in 6-well plates and maintained in a medium containing 10% FBS for 2 weeks. The number of colonies was counted after fixing and staining for 20 min.

### Cell invasion assay

The cell invasion assay was performed in 6.5-mm Transwell chambers (8-μm pore size; Millipore, MA, USA). A total of 2 × 10^5^ cells were seeded in the upper chamber coated with Matrigel for invasion assays. The upper chamber was filled with 200 μL of serum-free medium, while 800 μL of the medium with 20% FBS was added in the lower chamber. After incubation for 24 h, the cells on the filter surface were fixed and stained, followed by visualization using a phase-contrast inverted microscope.

### Tumor mouse model

Animal care and euthanasia were approved by the Nanjing Medical University Animal Studies Committee. Male athymic BALB/c mice (4 weeks old) were purchased from Lingchang company (Shanghai, China) and randomly divided into two groups. HCT116 cells and control cells (1 × 10^7^) stably knocked down for GAPLINC expression were implanted in the armpit area of 10 mice subcutaneously. The volume of the tumors was assessed every week after implantation. All mice were sacrificed after 4 weeks, and the xenografts were dissected and weighed. The tissues were fixed in 4% paraformaldehyde and embedded in paraffin, and then sectioned at 5 μm and stained with hematoxylin and eosin for histological and immunohistochemical analyses. The antibody Ki-67 (Abcam, MA, USA) was used for immunohistochemical analyses. The animal experimentation was reviewed and approved by the Nanjing Medical University Animal Studies Committee.

### Western blotting

Cell protein lysates were extracted from the logarithmically growing cells using radioimmunoprecipitation assay (RIPA) lysis buffer and separated by 10% sodium dodecyl sulfate–polyacrylamide gel electrophoresis (SDS-PAGE), and then transferred onto polyvinylidene difluoride membranes. After blocking in 5% bovine serum albumin for 1 h at 37^°^C, the membranes were incubated with various antibodies (Abcam, MA, USA) at 4°C overnight. Sequentially, the membranes were incubated with the secondary antibody, and the proteins were visualized via electrochemiluminescence.

### Microarrays and gene expression analysis

Total RNA was extracted from cells using TRIzol reagent (Invitrogen, Shanghai, China). The RNA integrity was assessed using standard denaturing agarose gel electrophoresis. Labeled samples were hybridized to the Agilent Human 4×44K Gene Expression Microarrays v2 (KangChen Biotech, Shanghai, China). After washing, the microarray slides were scanned using an Agilent DNA microarray scanner. The array images were analyzed by the Agilent Feature Extraction Software (version 11.0.0.1, CA, USA). Then, further bioinformatics analyses were performed using the Agilent GeneSpring GX software (version 12.1, CA, USA). Finally, pathway analysis and Gene Ontology (GO) analysis were performed to determine the roles of differentially expressed genes in these biological pathways or GO terms.

### RNA pull-down assay and mass spectrometry

The biotin-labeled RNAs of GAPLINC were *in vitro* transcribed using Biotin RNA Labeling Mix (Roche, CA, USA) and T7 RNA polymerase (New England Biolabs, MA, USA), and purified using the EZNA RNA Probe Purification Kit (Omega, GA, USA). The protein lysate from 10^7^ HCT116 cells were incubated with 2 μg of biotinylated RNA and mixed with T1 beads (Invitrogen, CA, USA). After washing five times in RIPA buffer, the RNA-bound proteins were retrieved and boiled in SDS buffer. Finally, the proteins were separated by SDS-PAGE and silver stained. Then, the particular bands were excised and detected by mass spectrometry analyses.

### RNA immunoprecipitation

The RNA immunoprecipitation (RIP) experiment was performed according to the manufacturer's instructions using the EZ-Magna RIP Kit (Millipore, MA, USA). Briefly, HCT116 cells were harvested and lysed with RIP lysis buffer. The PSF (Sigma, MO, USA), NONO (Abcam, Cambridge, MA, USA), and nonspecific control immunoglobulin G (IgG) antibodies were used for RIP. RIP lysates and antibodies bound to magnetic beads were incubated with rotation overnight at 4^°^C. Then, the proteins in the immunoprecipitate were digested, and the retrieved RNAs were detected by RT-PCR.

### Statistical analysis

A two-tailed Student *t* test was used to estimate the statistical significance of differences between the groups. A *P* value less than 0.05 was considered statistically significant, and the results were reported as mean ± standard deviation. Statistical analyses were performed using the SPSS software (version 18.0, New York, USA) and presented using the GraphPad prism software (version 6, CA, USA). All experiments were repeated at least three times.

## SUPPLEMENTARY FIGURE AND TABLES


