# Supplementary material for: Long noncoding RNA GAPLINC promotes invasion in colorectal cancer by targeting SNAI2 through binding with PSF and NONO
Source: Oncotarget. 2016 May 31;7(27):42183–94. doi: 10.18632/oncotarget.9741 (PMC5173126; doi:10.18632/oncotarget.9741)
Supplement: Supplementary file 1 [file oncotarget-07-42183-s001.pdf]

Long noncoding RNA GAPLINC promotes invasion in colorectal cancer by targeting SNAI2 through binding with PSF and NONO

SUPPLEMENTARY FIGURE AND TABLES

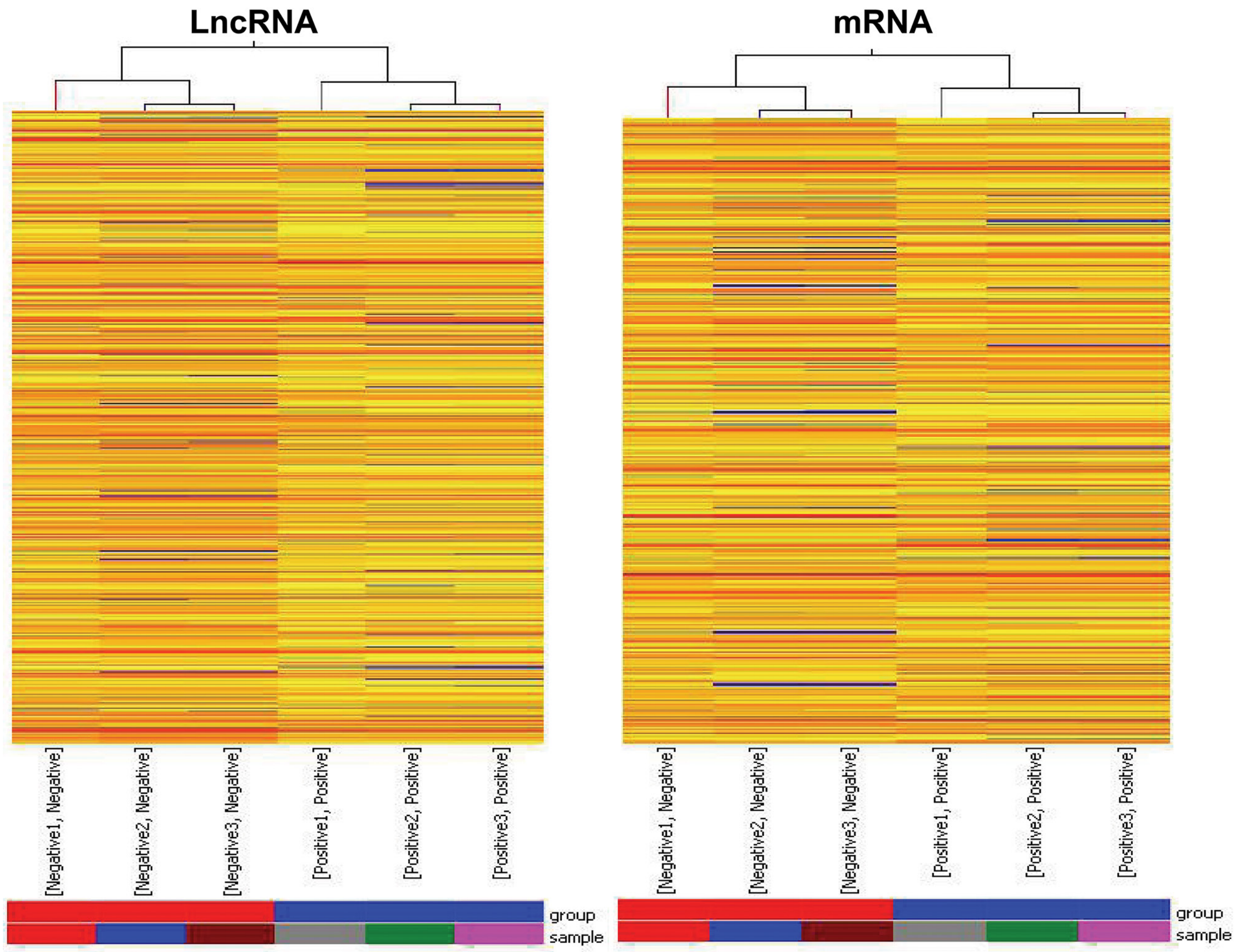

Supplementary Figure S1: Hierarchical clustering showing the differentially expressed lncRNAs and mRNAs in MLNs versus NLNs. Red indicates an increase and blue indicates low relative expression. Data represent mean ± standard deviation from three independent experiments. $*P < 0.05$, $**P < 0.01$.

Supplementary Table S1: Sequences information of the primers and siRNAs

Genes	Forward primer (5′–3′)	Reverse primer (5′–3′)
GAPLINC	ACACACAGCAGCCTGGTTTC	ATGGCACAATCAGGGCTCTT
PSF	CAAGATCTGATGAGACGCCAGGAA	CCATCTCACGTTGGCGAATCATCA
NONO	TCGGATGGGTCAGATGGCTA	CGGTTGAATGCAGGAGGAGT
LAMB3	GCCACATTCTCTACTCGGTGA	CCAAGCCTGAGACCTACTGC
SNAI2	TGGTTGCTTCAAGGACACAT	GTTGCAGTGAGGGCAAGAA
IGFBP7	CACTGGTGCCCAGGTGTACT	TTGGATGCATGGCACTCATAT
GAPDH	GCACCGTCAAGGCTGAGAAC	GGATCTCGCTCCTGGAAGATG
GAPLINC-siRNA1	GCCAUCUCCAUAACAUAAATT	UUUAUGUUAUGGAGAUGGCTT
GAPLINC-siRNA2	GCACUUAGAAGAACCAAUATT	UAUUGGUUCUUCUAAGUGCTT
PSF-siRNA	CUUUCUGUUCGUAAUCUUUCA	AAAGAUUACGAACAGAAAGGG
NONO-siRNA	GGGGUGGUAUUAAACAAGUCA	ACUUGUUUAAUACCACCCCUC

Supplementary Table S2: Pathway prediction using KEGG terms

Pathway ID	List of hits	*P* value	Pathway description
path:hsa05166	44	0.022717	Human T-cell lymphotropic virus I infection
path:hsa04932	35	0.000179	Nonalcoholic fatty liver disease
path:hsa04015	35	0.047893	Rap1 signaling pathway
path:hsa04062	33	0.028422	Chemokine signaling pathway
path:hsa03010	29	0.001971	Ribosome
path:hsa04514	29	0.006545	Cell adhesion molecules
path:hsa04310	26	0.022235	Wnt signaling pathway
path:hsa04660	25	0.000829	T-cell receptor signaling pathway
path:hsa04360	24	0.024384	Axon guidance
path:hsa04662	21	0.000127	B-cell receptor signaling pathway
path:hsa04640	21	0.002316	Hematopoietic cell lineage
path:hsa04064	20	0.007803	NF-kappa B signaling pathway
path:hsa04664	17	0.004824	Fc epsilon RI signaling pathway
path:hsa04520	17	0.007545	Adherens junction
path:hsa04612	17	0.016624	Antigen processing and presentation
path:hsa05210	14	0.018984	Colorectal cancer
path:hsa05221	13	0.02154	Acute myeloid leukemia
path:hsa05213	12	0.02442	Endometrial cancer
path:hsa03410	10	0.005552	Base excision repair
path:hsa04962	10	0.048117	Vasopressin-regulated water reabsorption
path:hsa00563	7	0.029704	Glycosylphosphatidylinositol-anchor biosynthesis
path:hsa00511	6	0.018586	Other glycan degradation

Supplementary Table S3: Proteins by GAPLINC pull-down and mass spectrometry analysis in HCT116

Hits	Protein mass (Da)	Protein description	Protein score
1	76216	Splicing factor, proline- and glutamine-rich OS=*Homo sapiens* GN=SFPQ PE=1 SV=2	978
2	59020	Keratin, type I cytoskeletal 10 OS=*Homo sapiens* GN=KRT10 PE=1 SV=6	865
3	54311	Non-POU domain-containing octamer-binding protein OS=*Homo sapiens* GN=NONO PE=1 SV=4	854
4	66170	Keratin, type II cytoskeletal 1 OS=*Homo sapiens* GN=KRT1 PE=1 SV=6	783
5	65678	Keratin, type II cytoskeletal 2 epidermal OS=*Homo sapiens* GN=KRT2 PE=1 SV=2	451
6	62255	Keratin, type I cytoskeletal 9 OS=*Homo sapiens* GN=KRT9 PE=1 SV=3	303
7	51872	Keratin, type I cytoskeletal 14 OS=*Homo sapiens* GN=KRT14 PE=1 SV=4	219
8	32060	Voltage-dependent anion-selective channel protein 2 OS=*Homo sapiens* GN=VDAC2 PE=1 SV=2	184
9	51163	Keratin, type I cytoskeletal 28 OS=*Homo sapiens* GN=KRT28 PE=1 SV=2	164
10	50788	Tubulin alpha-1A chain OS=*Homo sapiens* GN=TUBA1A PE=1 SV=1	159
11	60315	Keratin, type II cytoskeletal 6B OS=*Homo sapiens* GN=KRT6B PE=1 SV=5	158
12	48361	Keratin, type I cytoskeletal 17 OS=*Homo sapiens* GN=KRT17 PE=1 SV=2	140
13	95842	A-kinase anchor protein 4 OS=*Homo sapiens* GN=AKAP4 PE=1 SV=1	119
14	50095	Tubulin beta chain OS=*Homo sapiens* GN=TUBB PE=1 SV=2	117
